# Air leaks following pulmonary resection for lung cancer: is it a patient or surgeon related problem?

**DOI:** 10.1308/003588412X13171221592258

**Published:** 2012-09

**Authors:** H Elsayed, J McShane, M Shackcloth

**Affiliations:** ^1^Liverpool Heart and Chest Hospital NHS Foundation Trust,UK; ^2^Department of Thoracic Surgery, Ain Shams University,Egypt

**Keywords:** Air leak, Lung resection morbidity

## Abstract

**INTRODUCTION:**

Prolonged air leak (PAL) is the most common complication after partial lung resection and the most important determinant of length of hospital stay for patients post-operatively. The aim of this study was to determine the risk factors involved in developing air leaks and the consequences of PAL.

**METHODS:**

All patients undergoing lung resection between January 2002 and December 2007 in our hospital were studied retrospectively. Univariate analysis to predict risk factors for developing post-operative air leaks included patient demographics, smoking status, pulmonary function tests, disease aetiology (benign, malignant), neoadjuvant therapy (pre-operative radiotherapy/chemotherapy), extent and type of resection, and different consultant surgeons’ practice. A logistic regression model was used for multivariate analysis.

**RESULTS:**

A total of 1,911 lung resections were performed over the 6-year study period. An air leak lasting more than 6 days post-operatively was present in 129 patients (6.7%). This included 100 out of the 1,250 patients (8%) from the lobectomy group and 29 out of the 661 patients (4.4%) from the wedge/segmentectomy group. Using the multivariate analysis, the risk factors for developing an air leak included a low predicted forced expiratory volume in 1 second (pFEV_1_) (*p*<0.001), performing an upper lobectomy (*p*=0.002) and different consultant practice (*p*=0.02). PAL was associated with increased length of stay (*p*<0.0001), in-hospital mortality (*p*=0.003) and intensive care unit readmission (*p*=0.05).

**CONCLUSIONS:**

Air leaks after pulmonary resections were at an acceptable rate in our series. Particular patients are at a higher risk but meticulous surgical technique is vital in reducing their incidence. Our study shows that pFEV_1_is the strongest predictor of post-operative air leaks.

An air leak after pulmonary resection is almost always caused by an alveolar pleural fistula, which is defined as a communication between the pulmonary parenchyma distal to a segmental bronchus and the pleural space.^1^ Prolonged air leak (PAL) is reported to be the most common complication after lung resection. PAL results in a prolonged chest drain duration, which increases the risk of pleural infection, pulmonary embolism, respiratory distress and associated thoracic pain.PAL and associated co-morbidities prolong the post-operative length of hospital stay (LOS), affecting procedure related costs negatively. The aim of this study was to evaluate the proportion of air leaks after lung resections in our hospital over a six-year period as well as the risk factors associated with PAL and the interventions that can be performed to reduce this complication.

## Methods

All patients undergoing lung resection between January 2002 and December 2007 were included in the study. The hospital and theatre database as well as clinical notes were reviewed for information. This information was entered prospectively and reviewed retrospectively. Our database is 100% validated up to December 2007.

Pre-operative management included admission of patients on the day before the operation. A full pre-operative assessment was performed. Tumour staging was carried out using chest x-ray, computed tomography of the chest and abdomen, and positron emission tomography of the whole body. Routine blood and pulmonary function tests were performed. In patients with a cardiac history, echocardiography was also performed. In those with locally advanced resectable disease, neoadjuvant chemotherapy with/without radiotherapy was given.

Intra-operative management included a muscle sparing thoracotomy with single lung ventilation and a pulmonary or lobar/sublobar resection. We used a GIA™ 80mm stapler (Covidien, Mansfield, MA, US) or a TCT 75mm stapler (Ethicon, Somerville, NJ, US) to divide the fissures. The bronchus was stapled using a TLH 30mm stapler (Ethicon) unless there was a close resection margin where it was cut and hand sewn using multiple 3/0 PDS® sutures (Ethicon).

After resection the ipsilateral side was reconnected to the ventilator, and the bronchial stump and lung parenchyma were tested against 25cm water pressure using positive ventilation via hand bagging from the anaesthetist. Mild air leaks (with a ventilatory loss of <500ml/min of air) were treated conservatively while for moderate or severe air leaks the source was sought. If the bronchial stump was the cause, it was resutured and retested until no air leak was present. If the culprit was from the lung parenchyma, 4/0 Prolene® sutures (Ethicon) or Tisseel® biological sealant (Baxter, Deerfield, IL, US) was applied according to surgeon’s preference. Only a mild air leak was then accepted.

Post-operative management included routine application of low grade suction (5kPa) in Surgeon A’s practice only (consultant surgeons were labelled anonymously from A to H) while the routine for all other surgeons was keeping patients on water seal except for patients with a significant post-operative residual pleural space.

Patient demographics, respiratory co-morbidity, smoking status (pack-year history), pulmonary function tests (forced vital capacity, forced expiratory volume in 1 second [FEV_1_]), disease aetiology (benign, malignant), neoadjuvant therapy (pre-operative radiotherapy/chemotherapy), extent and type of resection, and different consultant surgeons were all included as factors causing PAL. PAL was defined as a leak for more than six days after lung resection.

Categorical variables were evaluated using a chisquared test for trend. The Kruskal–Wallis test was used for continuous variables. Potentially significant factors (*p*<0.1) were entered into the forward-stepwise logistic regression where a significance level of *p*<0.05 was required to remain in the model. Statisticalanalyses were conducted using MedCalc® (MedCalc Software, Mariakerke, Belgium).

## Results

### Patient demographics

Over the 6-year study period, there were 1,911 lung resections. There were 1,012 men (53%) and 899 women (47%). The median age of this population was 66 years (interquartile range: 57–73 years). Out of all resections, 1,250 were lobectomies (63.5%) and 661 wedge resections/segmentectomies (36.5%). Overall, 129 patients (6.7%) had PAL. Out of the lobectomy group, 100 patients (8.0%) had PAL and from the sublobar resection 29 (4.4%) suffered from PAL.

### Pre-operative factors

Among all the studied pre-operative factors, univariate analysis demonstrated that female sex (*p*=0.03), low body mass index (*p*=0.01), low predicted FEV_1_ (pFEV_1_) (*p*=0.0003), chronic obstructive pulmonary disease (COPD) patients on inhalers (*p*=0.001) and a higher pack-year history of smoking (*p*=0.03) were all significant factors in the development of PAL (Table 1). When these factors were entered into a multivariate analysis, only a low pFEV_1_ persisted as the most important predictive factor (*p*<0.0001) (Table 3).

**Table 1:** Results of univariate analysis for pre-operative patient characteristics

Patient characteristics	No air leak (*n*=1,782)	Prolonged air leak (*n*=129)	*p*-value
Women	852 (48%)	47 (36%)	**0.03**
Median age (IQR)	66 yrs (57–73 yrs)	66 yrs (58–72 yrs)	0.63
Median BMI (IQR)	26kg/m^2^ (23–29kg/m^2^)	25kg/m^2^ (21–28kg/m^2^)	**0.01**
Shortness of breath:*			0.73
0	500 (28%)	35 (27%)	
I	510 (29%)	32 (25%)	
II	589 (33%)	49 (38%)	
III	168 (9%)	12 (9%)	
IV	7 (0.4%)	0 (0%)	
Median FEV_1_ (IQR)	81 (66–95)	72 (57–90)	**0.0002**
Median FVC (IQR)	94 (80–107)	95 (81–109)	0.6
COPD	366 (21%)	41 (32%)	**0.003**
Smoking status			0.07
Current smoker	595 (30%)	48 (38%)	
Ex-smoker	1,159 (58%)	71 (56%)	
Non-smoker	249 (12%)	9 (7%)	
Median pack-year history of smoking (IQR)	30 (15–50)	40 (20–53)	**0.02**

IQR = interquartile range; BMI = body mass index; FVC = forced vital capacity; COPD = chronic obstructive pulmonary disease

*using New York Heart Association scale

**Table 2:** Results of univariate analysis for intra-operative variables characteristics

Disease and operative data	No air leak (*n*=1,782)	Prolonged air leak (*n*=129)	*p*-value
Extent of resection			**0.003**
Lobectomy	1,150 (65%)	100 (78%)	
Wedge resection	642 (35%)	29 (22%)	
Upper lobe resection	872 (43%)	88 (68%)	**<0.0001**
Benign disease	374 (19%)	12 (9%)	**0.008**
Pre-operative radiotherapy	185 (9%)	16 (12%)	0.23
Consultant			**<0.0001**
Surgeon A	505 (25%)	53 (41%)	
Surgeon B	69 (3%)	3 (2%)	
Surgeon C	168 (8%)	13 (10%)	
Surgeon D	492 (24%)	34 (26%)	
Surgeon E	80 (4%)	7 (5%)	
Surgeon F	80 (4%)	4 (3%)	
Surgeon G	20 (1%)	1 (1%)	
Surgeon H	564 (28%)	10 (8%)	
Surgeon I	37 (2%)	4 (3%)

**Table 3:** Significant factors for prolonged air leak remaining after logistic regression

Characteristic	Coefficient	Odds ratio	95% CI	*p*-value
Intercept	−1.8511			
Upper lobe resection	0.6788	1.97	1.28–3.04	**0.0002**
Lobectomy	0.6246	1.87	1.16–3.02	**0.01**
Consultant A	0.4305	1.54	1.03–2.35	**0.04**
Consultant H	−1.7696	0.17	0.08–0.37	**<0.0001**
Predicted FEV_1_	−0.0196	0.98	0.97–0.99	**<0.0001**

CI = confidence interval FEV_1 _= forced expiratory volume in 1 second Model c-statistic = 0.73 (showing an acceptable model fit)

### Intra-operative factors

**Figure 1:**
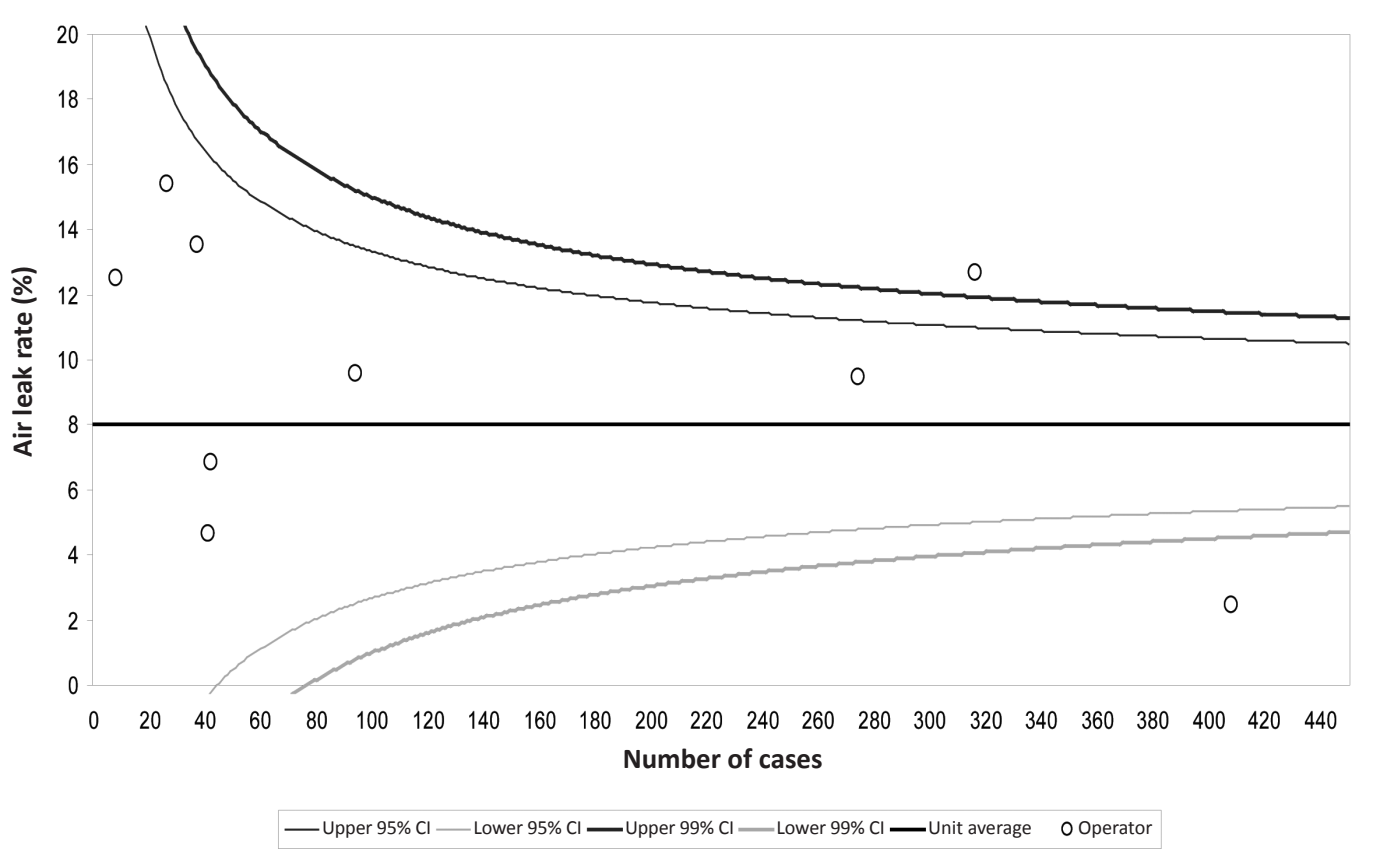
Incidence of air leak following lobectomy according to surgeons’ performance: Surgeon A is above the upper 99% confidence interval (CI) while Surgeon H is below the lower 99% CI.

Among all the studied intra-operative factors, a lobectomy was associated with PAL more than a sublobar resection (*p*<0.001). Resection of the upper lobe (*p*<0.001), malignant disease (*p*=0.008) and different consultant practice (*p*<0.001) were all significant factors in the univariate analysis (Table 2). When these factors were entered into a multivariate analysis, a lobectomy (*p*=0.001), resection of the upper lobe (*p*=0.0005) and different consultant practice were all significant factors for developing PAL. Consultant A had a significantly higher rate of PAL (*p*=0.04) and Consultant H had a significantly lower rate (*p*<0.0001) (Table 3). A funnel plot for the incidence of air leak according to surgeon’s performance is also shown in Figure 1.

### Clinical implications of PAL

The post-operative consequences of PAL included a median LOS of 7 days (IQR: 5–8 days) in patients with no air leak compared with a median of 13 days (IQR: 10–18 days) in the PAL group (*p*<0.0001). Intensive care unit (ICU) readmission was 5% in patients from the group with no PAL (*n*=82) and 9% in the PAL group (*n*=11) (*p*=0.05). In-hospital mortality was 2% (*n*=29) in the no PAL group and 6% (*n*=8) in the PAL group (*p*=0.003).

### Resource burden of PAL

The mean post-operative LOS for patients with no air leak was 7.9 days while the mean for PAL patients was 17.0 days. Over the study period time of 6 years, PAL was associated with a prolonged LOS quantifying up to 1,174 extra days or about 196 days per year (Table 4). (This does not include readmissions for drain removal following discharge with flutter bag in situ.)

**Table 4:** Burdens of prolonged air leak on length of hospital stay

	No air leak (*n*=1,782)	Prolonged air leak (*n*=129)	p-value
Median post-operative LOS (IQR)	7 days (5–9 days)	13 days (10–18 days)	**<0.0001**
Mean post-operative LOS	7.9 days	17.0 days	–
Total post-operative bed days	15,918	2,193	–

LOS = length of stay; IQR = interquartile range

### Surgeons’ difference in practice

Figure 1 shows the volume of lobectomy cases per surgeon and a funnel plot to demonstrate PAL incidence per surgeon. Surgeons A and H had the highest volume of lobectomy patients (63% and 80% respectively). Table 5 demonstrates the case mix per surgeon. Among the risk factors for PAL in our study there was no difference between surgeons’ patients regarding pFEV_1_(*p*=0.1) and upper lobe resection (*p*=0.2). Despite performing fewer lobectomies than Surgeon H (*p*<0.0001), Surgeon A had the highest incidence of PAL of 11% (*n*=53)(*p*<0.0001). Surgeon H had an incidence of 2% (*n*=10) and other surgeons 7% (*n*=66). This had no impact on the incidence of ICU readmission (*p*=0.92) or in-hospital deaths (*p*=0.87) between surgeons.

**Table 5:** Patient and operative characteristics and outcomes by surgical team (risk factors for prolonged air leak from our study are shaded)

Characteristic	Surgeon A (*n*=505)	Surgeon H (*n*=507)	Others (*n*=899)	p-value
Women	241 (48%)	260 (51%)	398 (44%)	**0.04**
Median BMI (IQR)	25kg/m^2^ (22–29kg/m^2^)	26kg/m^2^ (22–28kg/m^2^)	26kg/m^2^ (23–29kg/m^2^)	0.1
Median FEV_1_ (IQR)	83 (66–97)	81 (66–92)	79 (64–94)	0.1
COPD	104 (21%)	123 (24%)	180 (20%)	0.16
Current smokers	167 (33%)	167 (33%)	235 (26%)	**0.008**
Median pack-year history of smoking (IQR)	35 (18–50)	36 (17–53)	30 (10–48)	**0.0002**
Pre-operative radiotherapy	48 (10%)	34 (7%)	91 (10%)	**0.08**
Lobectomy	316 (63%)	408 (80%)	526 (59%)	**<0.0001**
Upper lobe resection	247 (49%)	272 (54%)	441 (49%)	0.20
Benign disease	104 (21%)	85 (17%)	193 (22%)	0.07
Prolonged air leak	53 (11%)	10 (2%)	66 (7%)	**<0.0001**
ICU readmission	25 (5%)	26 (5%)	42 (5%)	0.92
In-hospital deaths	9 (2%)	9 (2%)	19 (2%)	0.87

BMI = body mass index; IQR = interquartile range; FEV_1_ = forced expiratory volume in 1 second COPD = chronic obstructive pulmonary disease; ICU = intensive care unit

## Discussion

To our knowledge, this is the largest study looking at factors influencing air leak after lung resection in the current English literature. Studying the causes of air leaks may assist in finding possible methods to avoid or reduce this complication, which is a major cause of morbidity after lung resection. It is, therefore important to try to avoid this problem, in order to decrease LOS and prevent infection of the pleural space caused by the opening of the bronchial stumps, which is commonly associated with spillage of purulent material into the contralateral lung, and subsequent pneumonia, worsening this serious complication.^2^

We defined PAL as persistent air leak for more than six days after lung resection. We presume this is related to the previous average LOS for lung resection patients. There is, however, an arbitrary difference between centres in defining PAL. This issue was addressed by Adebenojo more than a decade ago.^3^In our experience, with the recent improvement in peri-operative care, the average LOS for lung resection patients has been reduced to around four days and we will amend our definition of PAL to persistent air leak for more than four days in our new database as, in our opinion, it could only be this complication keeping a patient in hospital after four days. Moreover, with the introduction of more video assisted thoracoscopic lobectomy cases, the definition of PAL should be revised again as the expected LOS should become even shorter.

Some author shave concluded that PAL increases complication rates after routine pulmonary resection.^4,5^Brunelli*et al* found an 8.2% to 10.4% rate of empyema in patients with air leak lasting more than 7 days versus a rate of only 0% to 1.1% in patients with lesser air leaks.^4^ However, they found no difference between the PAL patients and others for other cardiopulmonary complications. Varela *et al* found that air leak lasting at least five days was associated with greater pulmonary morbidity including atelectasis, pneumonia or empyema (relative risk: 2.78).^5^Okereke*et al* found that any air leak was associated with more complications (30% vs 18%,*p*=0.07).^6^ In the lung volume reduction population, post-operative complications also occurred more often in patients experiencing air leak (57% vs 30%,*p*=0.0004).^7^Our study indicates that PAL is associated with a longer hospital stay, an increased incidence of ICU readmission and a higher in-hospital mortality rate.

The increased financial burden on hospitals with PAL patients should be recognised. In our series, patients with PAL had an average extra LOS of about 196 days per year compared with patients with no PAL. Obviously, other factors may have contributed to the morbidity associated with these patients but this extra cost should be appreciated.

All studies of the association of LOS and/or costs with air leak after lung resection reported increased costs or LOS (or both) in patients with PAL.^4–9^ Varela*et al* found LOS to be increased by approximately 6 days at a total expense of more than €39,000.^5^Brunelli*et al* found LOS to be increased by 7.9 days.^4^Bardell and Petsikas found that PAL (defined as an air leak persisting for more than three days) increased LOS by four days and,of all factors studied,only PAL predicted increased LOS.^8^Irshad*et al* found that the three most frequent complications delaying discharge beyond post-operative day 5 were PAL, pulmonary infection and atrial fibrillation.^9^ In the lung volume reduction population (*n*=6), the mean LOS among survivors was 11.8 days in those with any air leak versus 7.6 days in those without an air leak (*p*=0.0005).

Identifying patients with a higher risk of PAL pre-operatively may be useful in counselling patients for a higher risk of prolonged hospital stay. It may also alert the surgeon to handle the lung more meticulously during surgery, mainly avoiding excess dissection in the fissures and trying to ensure that the patient leaves theatre with a minimal amount of air leak.

In our series, lobectomy was more likely to cause PAL than a wedge resection or a segmentectomy.In other series, upper lobectomy was a greater risk factor for developing post-operative PAL compared with smaller resections,^10^ presumably because they often result in a large apical air space with poor visceral–parietal apposition.Additionally, the right upper lobe anatomically abuts two fissures where the minor fissure is nearly complete in approximately 50% of cases.Consequently, there is more dissection in the fissure and hence a larger parenchymal raw surface area is exposed.

A low pFEV_1_ was the strongest pre-operative factor in developing PAL in our series. Reduced pulmonary function has been reported as one of the most consistent risk factors for PAL.^11–13^ COPD with a reduced FEV_1_is an important predictor of PAL because of the expression of an increased airway resistance and pathological parenchymal changes.

The three most important pre-operative risk factors for PAL in our series were lobectomy, upper lobe resection and reduced FEV_1_.Brunelli*et al*, however, published a risk score for PAL that included only reduced FEV_1_ and three other different factors.^14^ They found that significant and reliable predictors of PAL were FEV_1_<80%older age (>65 years), presence of pleural adhesions and low body mass index(<25.5kg/m^2^).

Although older age has never been reported to be a significant risk factor for PAL,^12^ elderly patients may have a more fragile lung parenchyma with a reduced healing capacity, which may predispose to the occurrence of this complication. The presence of important pleural adhesions has been found previously to be associated with this complication.^15^ Tears in the lung parenchyma may ensue during mobilisation of the lung and taking down of the adhesions. Finally, a low body mass index may be a marker of a poor nutritional status, which in turn may negatively influence the healing of the surgically damaged tissue. These factors should all be considered.

There was a significant variation between surgeons’ practice in our hospital regarding PAL. Surgeon A had the highest incidence of PAL while Surgeon H had the lowest. This is despite the fact that Surgeons A and H had the biggest volume of lobectomy cases (Fig 1). It can also be seen from Table 5 that among the pre-operative factors of increasing risk of PAL, there was no difference in surgeons’ patient mix regarding pFEV_1_or upper lobe resection. However, Surgeon A performed fewer lobectomies, which,according to our results,leads to a higher risk of PAL.

A probable explanation for the higher incidence of PAL in one of the surgeons’ practice was Surgeon A’s routine application of suction for post-operative patients while the rest of the surgeons used a water seal by default. Surgeon H, who had the fewest air leaks, routinely used sutures to seal them if anything more than a mild air leak was present after resection. He used no special glues or surgical seals. We believe that the routine application of suction can prolong air leak as it would delay parenchymal healing and facilitate the rupture of blebs/bullae, especially in patients with COPD. This hypothesis is also supported by a randomised controlled study performed by Cerfolio *et al*.^16^Nevertheless, PAL associated with Surgeon A’s practice was not associated with higher ICU readmission or in-hospital mortality.

### Limitations of the study

Although the number of cases was large, this was a single-centre clinical study that relied on retrospective review of data. In addition, quantification of air leak was not performed, precluding its use in analysis. Intra-operative findings of completeness of fissures were not recorded. This will be updated in our new database. Furthermore, the details of the post-operative management of PAL and its implications should probably be included in a more detailed study.

## Conclusions

Air leak after pulmonary resection was at an acceptable rate in our series. Particular patients are at a higher risk of PAL but meticulous surgical technique and avoiding the use of routine suction are significant factors in reducing the incidence of this complication. Our study shows that a low pFEV_1_is the strongest predictor of PAL and this group of patients with COPD should be carefully counselled pre-operatively.
